# Overexpression of ubiquitin specific proteases 44 promotes the malignancy of glioma by stabilizing tumor-promoter securin

**DOI:** 10.18632/oncotarget.16447

**Published:** 2017-03-22

**Authors:** Yongxiang Zou, Guanzhong Qiu, Lei Jiang, Zheng Cai, Wei Sun, Hongkang Hu, Chengyin Lu, Weilin Jin, Guohan Hu

**Affiliations:** ^1^ Department of Neurosurgery, Changzheng Hospital, Second Military Medical University, Shanghai, PR China; ^2^ Department of Neurosurgery, General Hospital of Jinan Military Command, Jinan, PR China; ^3^ Institute of Nano Biomedicine and Engineering, Department of Instrument Science and Engineering, Key Laboratory for Thin Film and Microfabrication Technology of Ministry of Education, School of Electronic Information and Electronic Engineering, Shanghai Jiao Tong University, Shanghai, PR China

**Keywords:** glioma, USP44, securin, cell cycle

## Abstract

Ubiquitin specific peptidase 44 (USP44) has been identified as an important component of spindle assemble checkpoint (SAC) to prevent the formation of aneuploidy. However, recent study raised a controversy about the effect of USP44 in tumor. Here, we first confirmed the intranuclear localization of USP44 by testing several specific antibodies to recognize endogenous USP44. Then, data from IHC and qRT-PCR assay indicated that the high expression of USP44 existed in high-grade glioma tissues and signified a poor prognosis. Knockdown of USP44 inhibited proliferation, migration and invasion, induced apoptosis, and arrested cell cycle in G2/M phase in the established glioma cell lines. Down-regulation of oncoprotein securin was detected in USP44 deficient cells, and the interaction of endogenous USP44 and securin was confirmed by immunoprecipitation in U251MG cells, which indicated that securin was a substrate of USP44, and might be stabilized by USP44. *In vivo*, knockdown of USP44 inhibited the tumorigenicity of U87MG cells significantly. Consequently, our findings suggested that overexpression of USP44 could enhance the malignancy of glioma via securin. USP44 might serve as a predictive biomarker, and the USP44-securin pathway might provide a new therapeutic strategy for the treatment of glioma.

## INTRODUCTION

Gliomas are the most common adult primary malignant tumors derived from the central nervous system (CNS), which exhibit a higher mortality rate than any other encephalic tumors [1]. Recent researches on glioma treatment have made considerable progress from the perspectives of epigenetic regulation, tumor metabolism, glioma stemness, and etc. However, these latest findings do not significantly improve patient's outcome nor apply to clinic broadly [2–4]. Under this circumstance, the molecular mechanism of gliomagenesis still needs to be investigated.

Ubiquitin proteasome system is involved in intracellular signaling pathways through regulating protein stability. The dynamic balance between ubiquitination and deubiquitination guarantees the stabilization of certain protein that is important to physiological and pathological processes [5]. Ubiquitin specific proteases (USPs) are an important group of deubiquitinating enzymes (DUBs), which consist of 56 members in human [6]. Many USPs participate in the process of tumorigenesis by stabilizing the tumor-related proteins [7]. USP9X can stabilize MCL1 by removing the Lys 48-linked polyubiquitin chains, which normally mark MCL1 for degradation and thereby promote tumor cell survival [8]. Down-regulation of USP28 resulted in LSD1 destabilization, leading to the suppression of cancer stem cell (CSC)-like characteristics *in vitro* and inhibition of tumorigenicity *in vivo* [9].

USP44 has been reported to regulate cell cycle control, DNA double-strand damage response and other important biological processes [10, 11]. However, there is still controversy about the precise role that USP44 plays in cancer. Robyn et al. proposed the USP44 was a tumor suppressor in colorectal cancer (CRC), and Paul et al. reported that the rate of chromosome mis-segregation and aneuploidy increased significantly in USP44-null mice MEFs [12, 13]. On the other hand, some researchers suggested that USP44+ cancer stem cell subclones might contribute to VM formation and the aggressive behavior in breast cancer [14]

It is possible that USP44 serves as tumor promoter by deubiquitinating substrates of APC such as securin. Traditional theory believed that dephosphorylated securin is recognized by APC^Cdc20^ and then ubiquitinated for proteasome-dependent degradation, leaving free separase to separate homologous chromosomes [15]. Although some researchers argued whether securin is necessary for mitosis in certain cells, the fact regarding securin as oncoprotein is indubitable [16–18]. The high expression level of securin appears in breast cancer, lung cancer, and bladder cancer, and it is widely involved in malignant proliferation, invasion, and angiopoiesis [19–21]. According to the published data, securin also serves as a tumor-promoter in glioma, and the overexpression of securin in high-grade glioma heraldes the bad prognosis [18, 22].

In this study, we detected the expression level of USP44 in glioma using tissue microarray and qRT-PCR, and we verified the correlation between the expression level of USP44 and prognosis. We confirmed that USP44 is a potential tumor-promoter by investigating the impacts of USP44 on the malignancy in glioma cell lines. We comfirmed that the oncoprotein securin was a substrate of endogenous USP44, to our knowledge, for the first time, and USP44 has a potency to protect securin from ubiquitination and degradation. We propose that USP44 promotes the malignant progression of tumors by stabilizing oncoprotein in addition to its carcinogenic potential. Moreover, the newly identified USP44-securin pathway may facilitate the understanding of the pathogenesis of glioma and provide new insights for glioma therapy.

## RESULTS

### Antibody identification and intracellular localization of USP44

**Figure 1:**
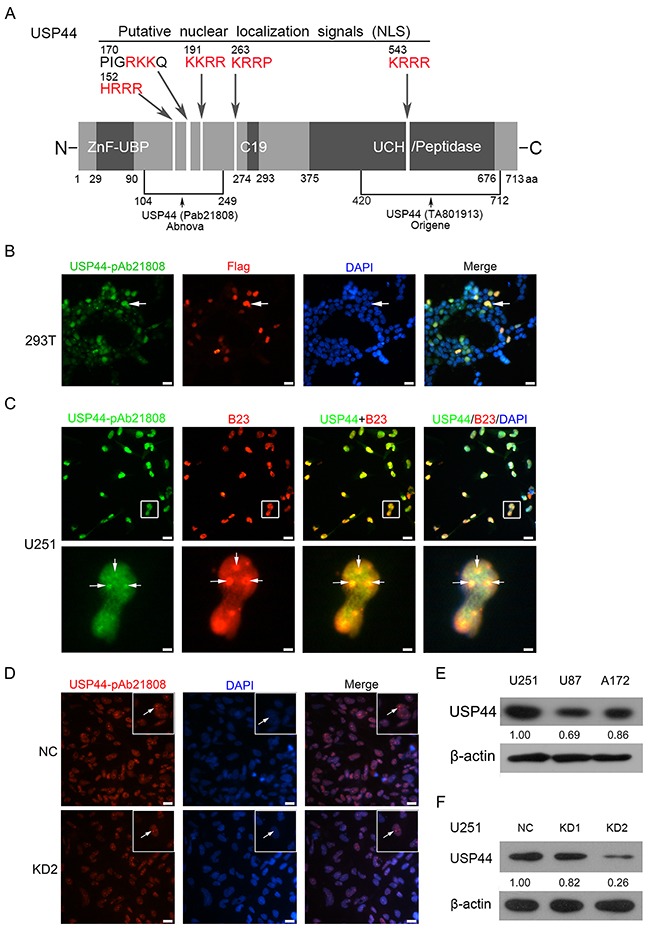
Antibody identification and intracellular localization of USP44 **(A)** A sketch map shows the fundamental structure of USP44 protein, the antibody-binding site and the potential nucleolus localization signal (red). **(B)** Flag-tagged USP44protein was expression in 293T cells, the IF signals of USP44 (green) and Flag (red) existed in same localization of nuclear. DAPI (blue) was used for nuclei staining. Scale bar, 50μm. **(C)** The co-localization of USP44 (green) and B23 (red) in nucleolus (blue) in U251MG cell was presented by immunofluorescence. DAPI was used for nuclei staining. Scale bar, 50μm/10μm. (D) IF assay analyzed expression of endogenous USP44 in U251-NC cell and U251-USP44-KD2 with an Alex Fluor 594 labeled secondary antibody (red), DAPI was used for nuclei staining, Scale bar, 50μm. **(E/F)** Expression of USP44 in glioma cell lines and the knockdown efficiency of two shRNA were tested by immunoblotting with antibody pAb21808.

Laboratory of Paul J. Galardy had verified several specific anti-USP44 antibodies, but none of them could detect endogenous USP44 by immunoblotting [13]. We examined the specificity of anti-USP44 antibodies recognizing different epitopes obtained from Abnova (pAb21808) and Origene (TA801913, 1D1). The antibody from Abnova detected endogenous USP44 more efficiently and specifically in both western blot and immunofluorescence assays. For illustration purpose, we included a schematic diagram here demonstrating the primary structural features of USP44 protein labeled with antibody combining sites and putative nuclear localization sites (Figure 1A). 293T cells were transfected with Flag-tagged USP44 protein. This fusion protein was detectable by immunofluorescence with both anti-USP44 antibody (Abnova pAb21808) and anti-flag antibody at the same position of cells (Figure 1B). Further, secondary antibodies labeled with FITC and Alexa-Fluor-594 were used to detect the accurate localization of endogenous USP44 and B23 (a nucleolus marker), respectivelyin U251MG cells. The signals of USP44 (green) and B23 (red) significantly overlapped in nucleolus, which indicates that USP44 shares the same subcellular localization with B23 in nucleolus (Figure 3C). Furthermore, the antibody (Abnova pAb21808) also performed excellently in detecting the difference of USP44 signal between U251-NC cells and U251-USP44-KD2 cells in immunofluorescence assay (Figure 1D). This specific antibody can also distinguish the expression level of USP44 in U251MG, U87MG, A172 and transfected U251MG cells by immunobloting (Figure 1E/1F). According to our data, we confirmed the specificity of the antibody in recognizing endogenous USP44 and the nucleolar localization of USP44. siRNA2# (KD2) presented a higher interferential efficiency than siRNA1# (KD1); therefore, we chose siRNA2# to perform the following experiments (Figure 1D/1F). The data of Origene (TA801913, 1D1) were shown as supplementary material (Supplementary Figure 1).

**Figure 2:**
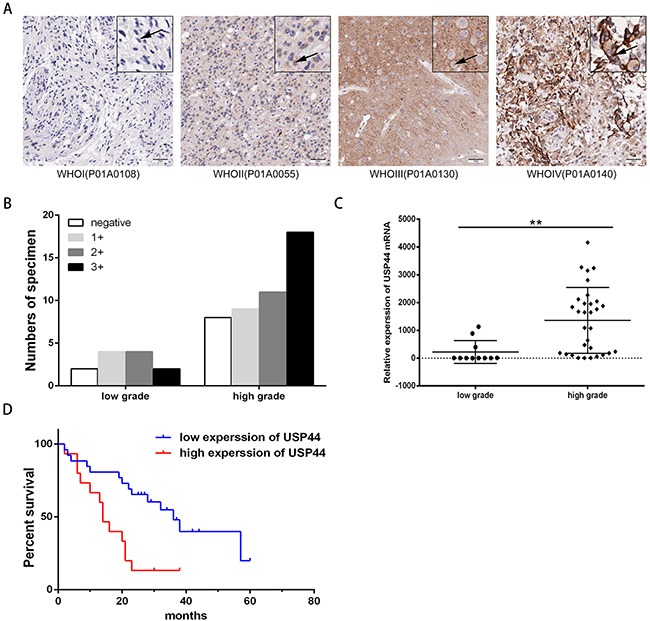
Protein and mRNA expression level of USP44 in glioma tissue samples **(A)** Representative IHC graphs of TMA presented the difference of USP44 expression and nuclear staining in different-grade glioma tissues. Scale bar: 50μm. **(B)** Histogram displayed the distribution of tissue samples with different score in low-grade and high-grade glioma respectively (p<0.05). **(C)** mRNA expression of USP44 was evaluated by qRT-PCR in 42 human glioma frozen tissue samples, there was a significant difference between low-grade glioma and high-grade glioma (**p<0.01). **(D)** Kaplan-Meier was used to analyze the discrepancy of overall survival caused by the expression of USP44 mRNA (p<0.05).

**Figure 3:**
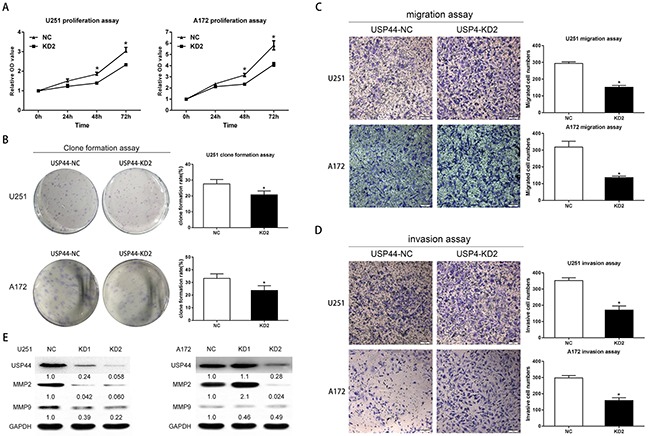
Knockdown of USP44 inhibit malignant biological behavior of glioma cells *in vitro* **(A)** Changes in cell proliferation were assessed by WST-1 after silencing USP44 in both U251MG (left) and A172 (right) cells (*p<0.05). **(B)** Clone formation assay was performed with U251-USP44-KD2, A172-USP44-KD2 cells and the corresponding NC cells. The statistical significance was presented in column chart on the right (*p<0.05). **(C/D)** Migration and invasion were tested withU251-USP44-KD2, A172-USP44-KD2 and corresponding NC cells by transwell assay, results were presented in histogram on the rightKnockdown of USP44 decreased the cell numbers in lower surface significantly (*p<0.05). Scale bar, 50μm. **(E)** Expression of MMP2 and MMP9 were probed in U251-USP44-KD1/2, A172-USP44-KD1/2 and corresponding NC cells by western blot.

### Protein and mRNA levels of USP44 are associated with the pathological grade and reduced overall survival in glioma

Next we analyzed the expression level of USP44 in glioma tissues using the specific antibody. TMA containing 61glioma specimens (three samples fell off) in different grades was used for immunohistochemistry (IHC) assay. As shown in the representative images, USP44-staining distributed both in nucleus and in cytoplasm, with a higher intensity in high-grade (WHOIII/IV) tissues than in the low-grade tissues (WHOI/II) (Figure 2A). The data of IHC was described as no expression (negative), weak expression (1+), moderate expression (2+), and strong expression (3+). Thus protein level of USP44 was increased in high-grade glioma samples simultaneously (p value of linear by linear association <0.05, Figure 2B).

Then, we examined 41 fresh-frozen glioma specimens of different gradesby qRT-PRC. The data indicated that the mRNA level of USP44 was significantly higher in 65.52 % of high-grade (WHOIII/IV) tissues than that in low-grade (WHO I/II) tissues, and the average multiple was 3.3. (**p <0.01 Figure 2C). The tissue samples were divided into two groups according to their average mRNA expression levels. The survival analysis revealed that patients with high mRNA expression of USP44 suffered a shorter overall survival (OS). (p <0.05, Figure 2D). Therefore, our data suggested that high expression level of USP44 was highly related to malignancy and bad prognosis of glioma.

### Depletion of USP44 inhibits proliferation, migration, and invasion in glioma cell lines

The result from tissue samples interested us in further study. We investigated the effect of USP44 to the established glioma cell lines with shRNA2# (KD2)

First, we assessed the proliferation of USP44-KD2 cells using WST-1 assay and clone formation assay. In WST-1 assay, the increasing range of OD450 reading in U251-USP44-KD2 and A172-USP44-KD2 cells was significantly decreased from 48 h to 72 h compared to the NC-cells (*p <0.05, Figure 3A). Clone formation ratio is another indicator of proliferation. Calculated results displayed smaller clone formation rate in U251-USP44-KD2 and A172-USP44-KD2 cells than in the NC cells (*p <0.05, Figure 3B). These results indicated that knockdown of USP44 inhibited the proliferation of glioma cells.

Then, migration and invasion were examined by transwell assay. In migration assay, more USP44-NC cells migrated to the lower face of the membrance than USP44-KD2 cells. Statistical results confirmed that knockdown of USP44 reduced the cell numbers migrating through the membrane in both U251-USP44-KD2 (151.3 ± 4.492) and A172-USP44-KD2 cells (136.0 ± 5.132), comparing to UPS44-NC cells (U251-NC: 293.5 ± 4.056; A172-NC: 318.3 ± 20.02). (*p <0.05, Figure 3C). In invasion assay, a statistically significant difference of invasion cell numbers occurred between the USP44-KD2 (U251-USP44-KD2: 170.8 ± 11.21; A172-USP44-KD2:157.7 ± 7.342) and the USP44-NC cells (U251-NC: 352.3 ± 10.17; A172-NC: 297.8 ± 6.019). (*p value <0.05, Figure 3D). The average proportions of the invaded cells of two groups were 48.5% and 52.9%, in U251and A172 cells, respectively. In addition, we detected matrix metalloproteinase-2(MMP2) and matrix metalloproteinase-9(MMP9) by immunobloting. The blot density of MMP2 and MMP9 declined drastically in U251-USP44-KD1 cells and U251-USP44-KD2 cells, comparing to U251-NC cells. Regarding A172 cells, the blot density of MMP2 and MMP9 in A172-USP44-KD2 cells was also in good agreement with the result from the invasion assay (Figure 3E).

Overall, the difference derived from our experimental results indicated that the knockdown of USP44 inhibited proliferation, migration, and invasion in glioma cells.

### Down-regulation of USP44 promotes the apoptosis of glioma cells *in vitro*

**Figure 4:**
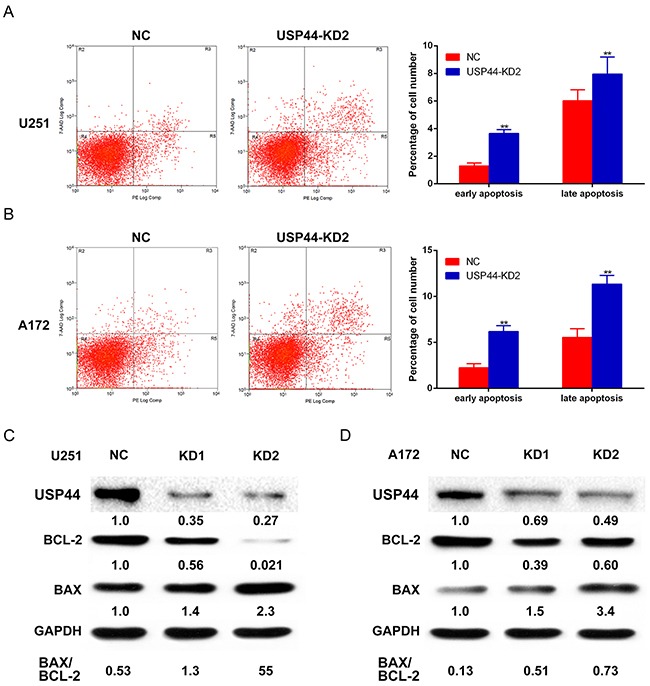
Knockdown of USP44 induces apoptosis in glioma cells **(A)** U251-USP44-KD2 cell and NC cell stained with PE and 7-AAD were analyzed by flow cytometry, the difference percentage of early and late apoptosis was displayed in the right histogram (**p<0.01). **(B)** A172-USP44-KD2 cell and NC cells were analyzed as mentioned above; the data was shown in histogram on the right(**p<0.01). **(C/D)** Apoptosis-related proteins (Bcl-2, BAX) were detected by western blot with specific antibodies and β-actin was served as the control of sample loading. The rate of BAX/BCL-2 was presented under the blotting.

The abnormal proliferation and clone formation presented in USP44-KD cells inspired us to further investigate the potential mechanism for USP44affecting proliferation. Apoptosis is a crucial mechanism that affects cell proliferation. The degree of apoptosis was represented in form of total percentages of annexin-positive populations that included the early apoptosis (PE-AnnexinV positive/7-AAD negative) and the late apoptosis (PE-AnnexinV positive/7-AAD positive). In U251-USP44-KD2 cells, the rates of early apoptosis (3.65± 0.28%) and the late apoptosis (7.28± 2.07%) were significantly higher than that in U251-NC cells (early: 1.12± 0.12%, late: 6.02± 0.79%; *p <0.05) (Figure 4A). A similar result was observed in A172-USP44-KD2 cells with the rate of early apoptosis (6.17± 0.64%) and the late apoptosis (11.32± 0.95%), compared to A172-NC cells (early: 2.23±0.46%, late: 5.55±0.94%; **p <0.01) (Figure 4B).

Further, we analyzed the changes of Bcl-2 and Bax by western blot as well. The blot density of Bax increased, while the blot density of Bcl-2 decreased in both U251-USP44-KD2 cells and A172-USP44-KD2 cells, which means down-regulating USP44 increased the ratio of Bax/Bcl-2 (Figure 4C/4D). Altogether, our results indicated that USP44 is an important factor in apoptosis regulation in glioma cells. The other immunobloting results of apoptosis-associated protein, such as PARP1 and caspase-3, are shown in the supplementary results (Supplementary Figure 1).

### Knockdown of USP44 arrest cell cycle at G2/M phase and affect the expression of cell cycle-associated proteins

**Figure 5:**
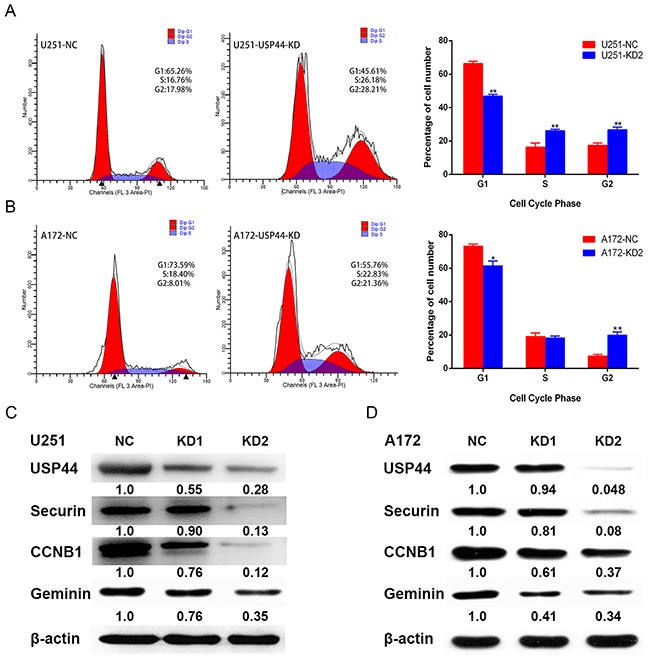
Down-regulation of USP44 arrest cell cycle in G2/M phase **(A/B)** Cell cycle distribution was analyzed by flow cytometry with PI-stainedU251-USP44-KD2 cells, A172-USP44-KD2 cells and corresponding NC cells. Difference of the distribution was shown in histograms on the right (*p<0.05, **p<0.01). **(C/D)** Expression of cell cycle-associated protein (CCNB1, securin, and geminin) were blotted by western blot with the specific antibodies, β-actin was served as the control of sample loading.

USP44 has been demonstrated to regulate cell cycle through antagonizing APC-induced ubiquitylation. Cell cycle is also another important regulation mechanism of proliferation. Hence, we investigated whether knockdown USP44 affects cell cycle distribution in glioma cells using flow cytometry. Our data showed that downregulating USP44 reduced the cell proportion in G0/G1 phase and increased cell proportion in S and G2/M phase in U251-USP44-KD2 cells (Figure 5A), the same trend also existed in A172-USP44-KD2 cells except the S phase (Figure 5B). There was a statistical significance in the difference of cell cycle distribution (*p <0.05, **p <0.01).

To further investigate the regulation mechanism of G2/M phase arrest, we analyzed the expression of typical G2/M-associated proteins, such as CCNB1, Securin, and Geminin. The blotting density of these proteins were significantly decreased in U251-USP44-KD1 and U251-USP44-KD2 cells, comparing to U251-NC cells (Figure 5C). The similar results occurred in the A172-USP44-KD1 and A172-USP44-KD2 cells (Figure 5D). These results suggested that knockdown of USP44 might inhibit glioma cell proliferation by arresting cell cycle in G2/M phase.

### Securin is the catalytic substrate of USP44, knockdown of USP44 promotes degradation of securin

It has been reported that USP44 inhibits the proteasome-dependent degradation of CCNB1 by hydrolyzing its ubiquitin chain [10]. Moreover, CCNB1 and securin were both the substrate of APC/C, therefore, we hypothesized that securin might be another direct substrate of USP44.

**Figure 6:**
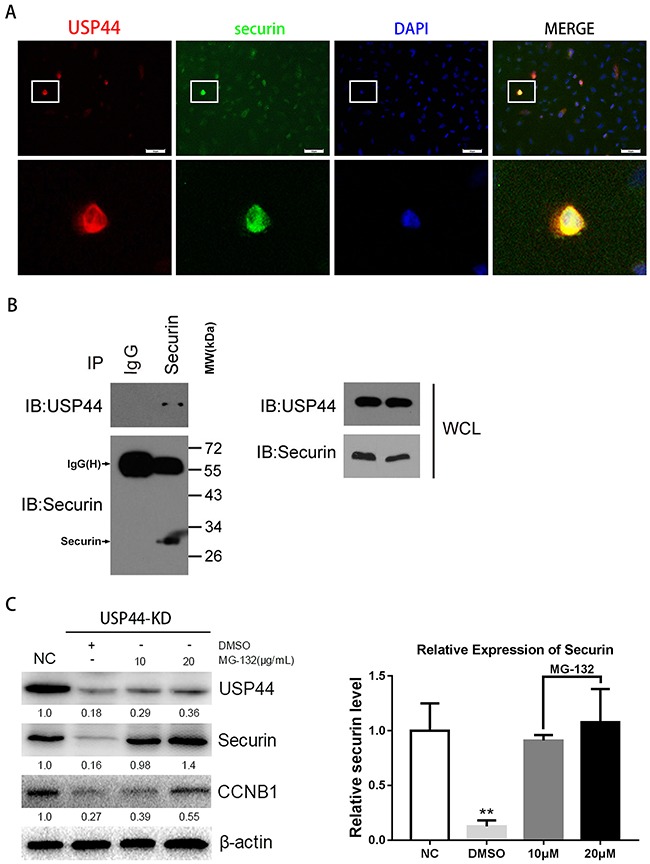
Interaction of endogenous USP44 and securin **(A)** Immunofluorescence signals of USP44 (green) and securin (red) were overlapped in U251MG cells. DAPI was used for nuclei staining. Scale bar, 50μm. **(B)** Sample was collected from abundant U251MG cells for immunoprecipitation with anti-securin antibody. IP sample was analyzed by western blotting with antibodies against the USP44 and securin. **(C)** The samples from U251-USP44-KD2 cells treated with 10μg/ml, 20μg/ml MG-132 (inhibitor of proteasome) or equal DMSO were analyzed by western blotting with the antibodies against securin and CCNB1. The relative level of securin was shown in histogram on the right (**p<0.01).

To confirm the hypothesis, we firstly performed immunofluorescence, immunoprecipitaion and western blot assays with U251MG cells. In immunofluorescence assay, the representative photograph shows an apparent overlap of USP44 and securin IF signal in nuclear division cells (Figure 6A), indicating the interactional probability exists between USP44 and securin. Next, immunoprecipitation assay was performed to confirm the potential interaction. Endogenous securin was immunoprecipitated from whole-cell lysates using the specific anti-securin antibody, and then IP sample was analyzed by immunoblotting assay with the antibodies against securin and USP44. Specific anti-IgG(H) secondary antibody was used to avoid the interference of IgG(L). The assay results showed that securin directly co-precipitated with USP44 (Figure 6B). Then, U251-USP44-KD2 cells were treated with proteasome inhibitor MG132, and protein level of securin was detected the by western blot. The blotting density of securin in MG132 (10μM and 20μM) treated cells was much higher than that in the untreated (NC and DMSO) cells (Figure 6C). A statistical significance of relative expression level existed among the four groups (**p <0.01). The results indicated that securin was another direct substrate of USP44, and USP44 might stabilize securin by inhibiting its ubiquitin-dependent degradation in glioma cells.

### USP44 knockdown inhibits tumorigenicity *in vivo*

**Figure 7:**
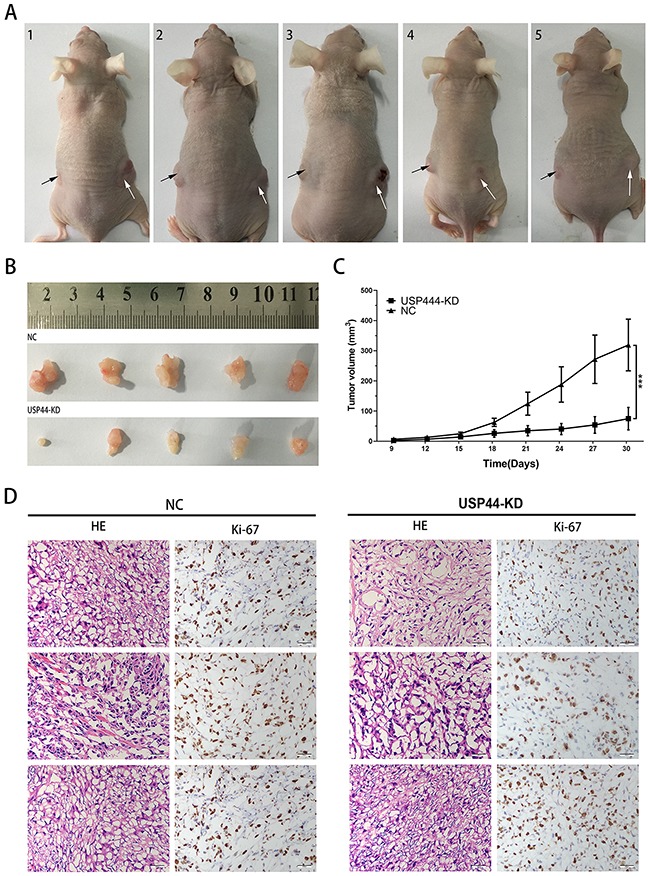
Downregulating USP44 inhibited the tumorigenesis *in vivo* **(A)** The photograph presented all nude mice model, the white arrow points to the tumor originated in U87-USP44-NC cells and the black arrow points to the tumor originated in U87-USP44-KD2 cells. **(B)** 30 days after subcutaneous injection, all nude mice were sacrificed and tumor tissues were sampled. **(C)** There was a statistically significant difference between the two group data of tumor volume (**p<0.01). **(D)** Excised tumor tissues were stained with HE and Ki-67. Representative photos were taken in 40 × magnification. Scale bar, 200μm.

Finally, we performed xenograft assay to evaluate the effect of USP44 in the process of tumor formation *in vivo*. Difference of tumor sizes on both sides of the mice was observed, the black array and the white array pointed to the tumor originated from U87-USP44-KD2 cells and U87-NC cells respectively (Figure 7A). The final volume of xenograft originated in U87-USP44-KD2 cells (74.96±37.27mm^3^) was significantly smaller than that in U87-USP44-NC cells (319.09±85.63mm^3^) (Figure 7B **p <0.01). In contrast to the tumors formed by U87-USP44-NC cells, the growth rate of tumors induced by U87-USP44-KD2 cells was apparently restricted with a statistically significant difference (***p value <0.001; Figure 7C). Then the paraffin sections of tumor were stained with H&E for histological assessment. The IHC were performed with anti-Ki-67 antibody. The morphological appearance of cells and the high staining intensity of Ki-67 suggested that the xenograft histogenesis was consistent with the cells inoculated (Figure 7D). The results from our xenograft experiment indicated that knockdown of USP44 can inhibit tumorigenicity of glioma cells *in vivo*.

## DISCUSSION

USP44 had the potential to affect the proliferation, invasion, apoptosis and cell cycle of somatic cell, but there is still a controversy about the specific effect of USP44 in malignancy. Zhang et al reported that USP44 expression level was elevated in subset of T-cell leukemia, but it is not clear whether this observation occurs in other tumors [23]. USP44 (+) CSC subclones may contribute to the prediction of VM formation and aggressive behavior [14]. Ohter studies highlighted tumorigenesis that is caused by deletion of USP44 in non-tumor cells while ignored the effect of USP44 in tumor cells [13]. One of the major causes of this controversy is lacking efficient antibody to recognize endogenous USP44. In our research, we identified several anti-USP44 antibodies that were suitable for immunofluorescence, immunohistochemistry, or western blot assays. Three lines of evidence validated the specificity of the antibody. First, the antibody specifically recognized single bands of 81 kDa USP44 as predicted by western blotting the cell lysates. Second, 293T cells were transfected with FLAG-tagged USP44, and the fluorescence signal of USP44 and flag existed in the same compartment. Third, knockdown of USP44 by shRNA drastically reduced the signals of both immunofluorescence and Western blot. Using the specific antibody, we validated the intracellular distribution and nucleolar localization of USP44 more precisely in glioma cells. These results are consistent with our prediction as well as the data from Human Protein Atlas (FLJ14528).

With the specific antibody, we analyzed the expression and effect of USP44 in glioma for the first time. Data from TMA and qRT-PCR confirmed that USP44 was highly expressed in high-grade glioma tissues compared to the low-grade glioma tissues. The high expression of USP44 was also associated with the poor prognosis according to the survival analysis. In established glioma cell lines, knockdown of USP44 inhibited the proliferation, migration, and invasion of the cells. Meanwhile, downregulating USP44 also induced apoptosis. In light of our experimental results and previous studies, we suggested that knockdown of USP44inhibited the malignancy of tumor cells, while promoting the transformation of non-tumor cells. Zhao et al reported that KLF8 promoted proliferation in the human ovarian cell by up-regulating CCND1 and down-regulating USP44 expression [24]. The different effect of USP44 in tumor cells and non-tumor cells reflects the complexity of the molecular regulation mechanism in tumor pathogenesis. In different molecular environments, the effect of single molecule on signal pathways may be different, except for its putative function.

Participating in regulation of cell cycle is an important function of USP44. Previous researches have demonstrated that numerous cell-cycle-associated proteins, such as cyclin D1, cyclin E, cyclin A, p21, p27, and p53, are the substrates of DUBs [25–29]. Our data showed that knockdown of USP44 arrested the cell cycle in G2/M phase concomitantly with the degradation of securin. Existing evidence verified that Cyclin B1 was a substrate of USP44, but the interaction between securin and USP44 was still unknown [10]. The result of immunoprecipitation assay confirmed the specific interaction of endogenous USP44 and securin for the first time. The proteasome inhibitor MG-132 could reverse the down-regulation of securin that was induced by deleting USP44, which means that USP44 stabilizes securin like other substrates by antagonizing the ubiquitin proteasome pathway. Securin prolonged cell cycle time by binding with separase until the anaphase onset [5]. However, knockdown of securin induced G2/M phase arrest in our experiment, but there is still no definite evidence to illustrate the accurate effect of securin on cell cycle in tumor cells. A single mutation in the one of the phosphorylatable residues of securin might be the reason for triggering the oncogenic properties of securin [30].

Intranuclear localization of proteins usually suggest their potential functions. Our experiment confirmed that USP44 had the same nucleolus localization with B23 as USP36, which means the two proteins might share a similar function. The proliferation of USP36-depleted cells was severely inhibited as a result of deficient nucleolus function and ribosome biogenesis anomaly [31]. Analogously, the rate of proliferation in glioma cells was also reduced by downregulating USP44. Moreover, USP44 affects the intranuclear dynamic mono-ubiquitylation alternation of H2Bub1 whose function was related with transcription and DNA damage response [32]. Manipulating ubiquitination degradation of nucleolar proteins may be another mechanism of USP44 to affect cell proliferation in glioma cells. However, the influence of USP44 on ribosome synthesis in human malignant glioma still needs further investigation.

## MATERIALS AND METHODS

### Tissue microarray and glioma tissue samples

The tissue microarray (Bra-Gli065PG-01) was purchased from Shanghai Outdo Biotech Company (Shanghai, China). The samples of TMA were obtained from Biochip National Engineering Research Center of Shanghai, the details were shown in Table 1. 42 fresh-frozen tissue samples of primary human glioma were collected from Neurosurgery Department of Shanghai Changzheng Hospital (Shanghai, China) between 2012 and 2014 with the promise of patients, the clinical and pathological data were shown in Table 2. All the samples were classified according to the WHO standard (David N. Louis et al. 2007).

**Table 1:** Clinico-pathological features of 61 patients of glioma consist in the tissue microarray

Variant	Patients numbers (%)
Age	
≥40	52 (85.26%)
<40	9 (14.74%)
Gender	
Male	30 (49.18%)
Female	31 (50.82%)
Tumor grade	
I/II	12 (19.67%)
III/IV	49 (80.33%)
Tumor position	
Frontal lobe	29 (47.54%)
Parietal lobe	7 (11.47%)
Temporal lobe	15 (24.59%)
Occipital lobe	2 (3.28%)
Ventricle	3 (4.92%)
Corpus callosum	2 (3.28%)
Thalamus	2 (3.28%)
Cerebellum	1 (1.64%)

**Table 2:** Clinico-pathological features of 41 patients of glioma for qRT-PCR

Variants	Tissue samples	USP44		P value
		High	Low	
Age				
≥40	34	14	20	0.183
<40	7	1	6	
Gender				
Male	25	8	17	0.332
Female	16	7	9	
Tumor size				
≥5cm	24	9	15	0.575
<5cm	17	6	11	
WHO grade				
I/II	13	1	12	0.009
III/IV	28	14	14	
KPS				
≥70	37	13	24	0.467
<70	4	2	2	

### Immunohistochemistry assay

Dewax the tissue microarray(TMA) and hydrate it in graded alcohol series. After epitope retrieval, the prepared array was incubated with anti-USP44 antibodies (1:50 dilution, abnova, pab21808), followed by incubation with horseradish peroxidase-conjugated secondary antibody (1:1000 dilution, proteintech). Then, diaminobenzidine (DAB) histochemistry Kit (Thermo scientific, 34065) was used forstaining... The percentage of positive tumor cells was scored as 0=0%-4%, 1=5%-25%, 2=26%-50%, 3=51%-75%, 4=76%-100%. The staining intensity was scored as 0 = negative, 1 = weak, 2 = moderate and 3 = strong. The final grading were the arithmetic product of percentage and staining intensity scores [(-) =0, (+) = 1~4, (++) = 5~8, (+++) = 9~12].

### RNA isolation and qRT-PCR

Total RNA (500ng) of each fresh-frozen sample was extracted in enzyme-free condition by using TRIzol Reagent (Invitrogen, 15596026), then transcribed into cDNA with the Prime Script™ RT reagent Kit (Takara, RR037A). The quantitative real-time PCR reactions were performed with SYBR^®^ Premix Ex Taq™ Kit (Takara, RR420A) in ABI 7900HT PCR instrument (Applied Biosystems, USA). The USP44 primers were, 5’-AACATGGTTTGAACAATCACCCA-3’ (forward) and 5’-GAGCCCTTGTAAACGTAAACTCT-3’ (Reverse). The two-step program was 95°C for 30s, followed by 40 cycles at 95°C for 5s, and 60°C for 34s. The data were analyzed with SDS v2.4.1 software. All assays were repeated in triplicate.

### Cell culture and MG-132 treatment

The human glioma cell lines U251MG, A172 and U87MG were purchased from Chinese Academy of Sciences Cell Bank (shanghai, china) [33]. U251MG and A172 cells were cultured in high glucose DMEM (Hyclone, SH30243.01B) with 10% Fetal Bovine Serum (Gibco, 10099141), meanwhile the U87MG cells was cultured in Eagle's MEM (Hyclone, SH30024.01B) with 10% Fetal Bovine Serum (Gibco, 10099141). All cells were incubated at 37°C in a humidified thermotank containing 5% CO_2_. For MG-132(TOCRIS, R&D Systems) treatment, adding MG-132 (dissolved in DMSO) into culture medium diluting to a final concentration of 10μM and 20μM, then culturing and culturing for 6 hours.

### Lentivirus transfection and puromycin treatment

The construction of lentiviral vectors (hU6-MCS-Ubiquitin-EGFP-IRES-puromycin) were entrusted to GeneChem Co. Ltd (Shanghai, China). The target sequences for USP44 shRNA 1# was GAACAUGGUUUGAACAAUC, for USP44 shRNA 2# was GCACAGGAGAAGGATACTAAT, and for shRNA-NC was TTCTCCGAACGTGTCACGT. U251MG, A172 and U87MG cells cultured in six-well plates for 24h were transfected with lentivirus. 8 hours later, replace the culture medium with fresh medium. 48 hours later, subculture the transfected cells into new 60mm dishes in a ratio of one over ten with 1.5μg/ml puromycin (ThermoFisher, A1113803). Continuous culture the cell until the stable transfected cell strains were obtained.

### Western blotting and antibody

Extract the whole cell lysate with ice-cold RIPA lysis buffer(20 mM Tris-HCl (pH 7.5), 150 mM NaCl, 1mM Na2EDTA, 1 mM EGTA, 1% Triton, 2.5 Mm sodium pyrophosphate, 1 mM beta-glycerophosphate, 1 μg/mL leupeptin, and 1 mM PMSF). The protein samples were boiled at 100°C for 5 min with 5× loading buffer and then separated by 12% SDS-PAGE and electrically transferred to 0.45 μm PVDF membranes. The membranes were blocked in 5% skimmed milk dissolved in TBST for 1.5 h at room temperature, then incubated with first antibodies at 4°C overnight. The antibodies included USP44 (Abnova, PAB21808), CCNB1 (abcam, ab181593), securin (Abcam, ab79546), caspase3 (Abcam, ab138502), Bcl-2 (Abcam, ab32124), BAX (Proteintech, 50599-2-ap), Geminin (Santa Cruz, sc-74456), PARP1 (Proteintech, 22999-1-ap), MMP2 (Proteintech, 10373-2-ap) and MMP9 (Proteintech, 10375-2-ap). Band intensity was detected using ChemiDoc XRS (Bio-Rad, USA) with Pierce™ ECL Western Blotting Substrate (ThermoFisher, USA).

### Immunoprecipitation

Whole cell lysate of normal U251MG cell (≥1×10^7^ cells) in 2,000ul RIPA lysis buffer was divided into two microcentrifuge tube equally, then incubated with anti-securin antibody and anti-IgG antibody(abcam, ab109489) at 4°C respectively. After 4 hours, adding protein A/G beads (Santa Cruz) to each microcentrifuge tube and incubating the mixed samples at 4°C overnight. The beads were precipitated by centrifugation at 8,000g (4°C, 30s) and washed thrice with 1ml RIPA lysis buffer. Proteins bound to the protein A/G beads were released by heated at 100°C for 5 min in the 2 × SDS loading buffer. The proteins were detected by western blot assay as previously mentioned.

### Cell proliferation and clone formation assay

Subculture the transduced cells into 96-well plates as a density of 1×10^3^ cells/well, each group had six parallel holes. Cell viability was tested with the WST-1 (Roche, 05015944 001) by measuring OD450 nm every 24 hours for 4 days. The data were normalized and all assays were performed in triplicate. In clone formation assay, the transfected cells were cultured in 6-well plates as a density of 500 cells/well. Terminate the culture when the cell clone was visible to the naked eyes, then fixed cells with 4% paraformaldehyde for 15 minutes followed by 0.05% crystal violet staining for 30min. Clone formation rate = (clone counts/500) x 100%.

### Migration and invasion assay

The migration assay was performed using 6.5 mm Transwell^®^ with 8μm pore polycarbonate membrane insert (Corning, CLS3422-48EA). Add 0.5 ml cell suspension (2.5×10^4^ cells) in serum-free culture medium into the upper chamber and 750μl culture medium containing 10 % FBS into the lower chambers. After 24 h, removing the cells on the upper surface of the membrane by scrubbing with cotton-tipped swabs quickly, and fixing the cells on the lower surface with pure methanol for 30 min followed by staining with analytical standard crystal violet (Sigma Aldrich, 32909) for 30min. Cell counting is facilitated by photographing the membrane through the microscope. For the invasion assay, 0.5ml cell suspension (2.5×10^4^ cells) in serum-free culture medium was added in the BioCoat™ Matrigel™ Invasion Chamber (BD, 354480), and the following procedures were in concordance with the migration assay.

### Immunofluoescence assay

Culture the transfected U251MG and A172 cells on TC-treated glass coverslips for 24 hours. The cells were rinsed with PBS, fixed with 4% paraformaldehyde for 15 min, and permeabilized with 0.5% Triton X-100 for 20 min. Blocking antigen with 5% skimmed milk for 60 min, the coverslips were incubated with rabbit polyclonalanti-USP44 (1:100) and mouse monoclonal anti-securin (Santa Cruz, 1:100) at 4°C overnight. Then co-incubated with Alexa Fluor^®^ 488-labeled goat-anti-rabbit and Alexa Fluor® 647-labeled goat-anti- mouse secondary antibody (Proteintech 1:200)at 37°C for 1h. DAPI (Life Technologies, 62248) was used to stain the nucleolus. The coverslips were photographed under fluorescence microscopy (BX53, Olympus, Japan).

### Cell cycle and apoptosis analysis by flow cytometry

At least 1 × 10^4^ transfected U251MG or A172 cells were rinsed with PBS, trypsinized and fixed in 70% pre-cold ethanol at 4°C overnight. Then cells were stained with 500μl PI/RNase Staining Buffer (BD, 550825) at room temperature for 15 min. Cell counts in different cell cycle phase were measured by CyAn ADP 9 colors flow cytometry (Beckman Coulter, USA) and the data were analyzed by Modfit LT 4.1v software. For apoptosis assay, 2 × 10^5^ transfected U251MG or A172 cells were trypsinized, and then co-incubated with Pharmingen™ PE Annexin V(BD, 554656) and 7-Amino-Actinomycin D (7-AAD)(BD, 559925) for 15 min at 4°C in lucifuge condition. After vortex for 15s, the ratio of apoptosis was measured by CyAn ADP 9 colors flow cytometry and the outcome were obtained via Summit V4.3 software.

#### Tumor xenografts

Subcutaneously inoculate 100ul monoplast suspension containing about 2×10^6^ U87MG-USP44-shRNA 2# transfected cells into the upside of the left hind limb of 5-week old male nude mice; suspension of U87MG-NC cells were inoculated into the right side. The animals were kept under pathogen-free sterile condition with sterile food and water. Tumor size was measured by caliper every three days. 30 days after injection, the mice were executed by cervical dislocation to obtain the tumor samples. The volume of tumor was calculated according to the formula: v=πab^2^/6 (a: major axis; b: minor axis).

### Statistical analysis

The percentage of samples with different IHC score in each grade of glioma was analyzed with Pearson χ^2^. Comparison of mRNA expression between low-grade and high-grade glioma tissues was performed using Cochran & COX t-test. Survival analysis was done by the Kaplan–Meier analysis. Data obtained from at least three independent experiments *in vitro* were analyzed by ANOVA. P value < 0.05 is considered to be statistically significant.

## SUPPLEMENTARY MATERIALS FIGURE



## Abbreviations

USP44: ubiquitin specific peptidase 44
CCNB1: cyclin B1
CNS: central nervous system
DUB: deubiquitinating enzyme
SAC: spindle assembly checkpoint
APC: anaphase promoting complex
DMEM: Dulbecco's Modified Eagle's Medium
MEM: minimum essential medium
TMA: tissue microarray
DAB: diaminobenzidine
PARP1: poly(ADP-ribose) polymerase 1.
