# Supplementary material for: Overexpression of ubiquitin specific proteases 44 promotes the malignancy of glioma by stabilizing tumor-promoter securin
Source: Oncotarget. 2017 Mar 22;8(35):58231–46. doi: 10.18632/oncotarget.16447 (PMC5601647; doi:10.18632/oncotarget.16447)
Supplement: Supplementary file 1 [file oncotarget-08-58231-s001.pdf]

Overexpression of ubiquitin specific proteases 44 promotes the malignancy of glioma by stabilizing tumor-promoter securin

SUPPLEMENTARY FIGURE

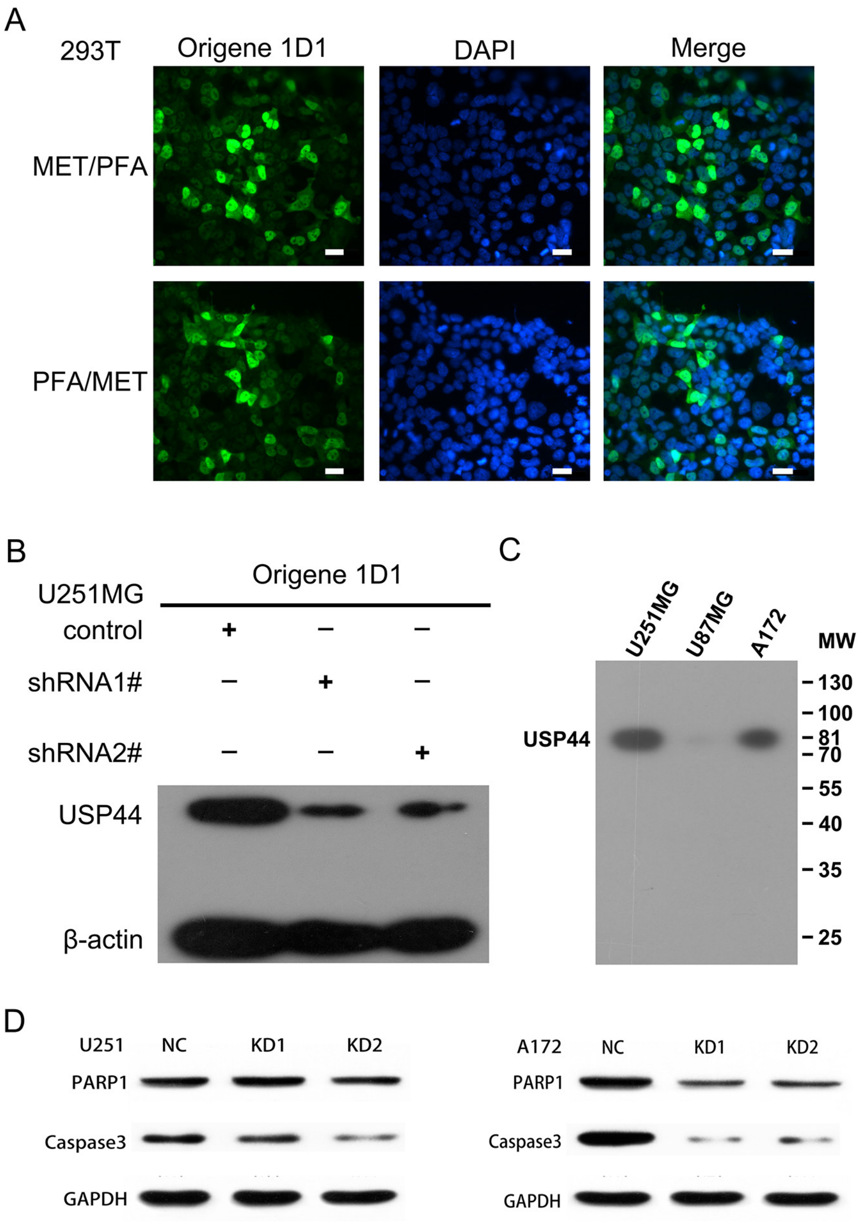

Supplementary Figure 1: Antibody Origene-1D1 can also recognize the endogenous USP44. (A) 293T cells were fixed by paraformaldehyde (PFA) and cold-methanol (MET) in two different orders, the endogenous USP44 was detected in nuclear by immunofluorescence with the antibody origene-1D1. **(B)** Samples from U251-USP44-KD1 cells and U251-USP44-KD2 cells were analyzed by immunobloting with the antibody origene-1D1. **(C)** Samples from U251MG, U87MG, and A172 cells were analyzed by western blotting with the antibody ABNOVA pAb21080. **(D)** Protein samples from USP44-KD2 cells and NC cells were examined by western blotting with specific antibodies of PARP1 and Caspase-3.
